# Gradient Systems and Asymmetric Relaxations in View of Riemannian Geometry †

**DOI:** 10.3390/e28050516

**Published:** 2026-05-02

**Authors:** Alessandro Bravetti, Miguel Ángel García Ariza, José Roberto Romero-Arias

**Affiliations:** 1 School of Science and Technology, University of Camerino, 62032 Camerino, Italy; alessandro.bravetti@unicam.it; 2 Instituto de Investigaciones en Matemáticas Aplicadas y en Sistemas, Universidad Nacional Autónoma de México, Ciudad de Mexico 04510, Mexico; roberto.romero@aries.iimas.unam.mx

**Keywords:** information geometry, gradient flows, asymmetric relaxations, non-metricity tensor, 53B12, 53B05, 53B50

## Abstract

In dually flat manifolds, there is a deep connection between gradient flows and pregeodesics. This was one of the many important contributions of Amari to information geometry. In this paper, we extend the study of this relationship to general Riemannian manifolds. Our result does not impose conditions of flatness on the connection or symmetry on its non-metricity tensor, thus broadening the geometric setting beyond Hessian manifolds. Within this framework, we provide a criterion for comparing relaxation along two different gradient descent curves of a function, formulated in terms of the non-metricity tensor of a connection for which the gradient curves are pregeodesics. We use it to study Gaussian chains, whose relaxation trajectories coincide with gradient descent curves in the space of Gaussian distributions. Thus, we recover a recent result that establishes a universal asymmetry: warming up is faster than cooling down. Our work illustrates how geometric insights rooted in Amari’s legacy offer new perspectives for optimization problems and stochastic processes.

## 1. Introduction

There is a classical result in mechanics, known as the Jacobi–Maupertuis Theorem, that relates the trajectories of a system to geodesics [1,2,3]. It states that, given a Riemannian manifold (M,g) and a mechanical Hamiltonian

$$H=\frac{1}{2}{\left||p|\right|}_{g}^{2}+V\left(q\right)$$

on $T^{*}M$, the projections to *M* of the trajectories of the Hamiltonian system at fixed energy *E* are reparametrized geodesics of the Jacobi metric

$$g^{J}=2\left(E-V\left(q\right)\right)g\;.$$

The Jacobi–Maupertuis theorem can be restated in terms of connections: the Hamiltonian trajectories at fixed energy are reparameterized geodesics of the Levi–Civita connection of the Jacobi metric $g^{J}$. Since the Hamiltonian vector field $X_{H}$ is the symplectic gradient of *H*, defined by

$$\omega (X_{H},\cdot )=dH\;,$$

the theorem provides a connection that turns symplectic gradient curves into geodesics.

Analogously, the Riemannian gradient of a function *f* is defined by

g(gradf,·)=df.

A natural question then arises: given a Riemannian manifold (M,g) and a function *f*, can we always find a connection that turns gradient curves into geodesics?

Fujiwara and Amari gave a partial answer to this question, showing that on dually flat (Hessian) manifolds, the gradient flow of a canonical divergence from a fixed point follows pregeodesics of the corresponding flat connection [4].

Despite the limitation of this result, coming from the assumption of dual-flatness, it has inspired continued interest, with recent applications spanning Weyl geometry, Hamiltonian dynamics, and non-equilibrium physics [5,6,7,8,9,10]. It has also oriented the quest to find canonical divergences on non-dually flat manifolds (see, e.g., [11] and the discussion therein).

Because the state space of a thermodynamic system is dually flat, we can use Fujiwara and Amari’s result to study asymmetric relaxations in this context. By asymmetric relaxations, we mean processes in which a system evolves faster from one initial state than from another to a common final equilibrium state, even when both initial states are equally distant from it [12,13,14,15,16]. Using the canonical divergence as the measure of distance to the final state, we showed that the faster relaxation curve is the one for which the Amari–Chentsov tensor evaluated along the curve is smaller whenever the speeds coincide [17]. This endowed the Amari–Chentsov tensor with a clear dynamical meaning, yet the result remained confined to the dually flat setting.

This naturally raises a second question: Can we generalize this criterion beyond dually flat manifolds?

Guided by these questions, we present two main results. First, we construct a connection of which the gradient curves of a prescribed function are pregeodesics. This extends Fujiwara and Amari’s theorem beyond dual flatness, and provides the foundation for the second result. Using this connection, we derive an asymmetry criterion valid on any Riemannian manifold, expressed in terms of the connection’s non-metricity tensor.

We illustrate the scope of this extended framework by applying it to recover the universal warming–cooling asymmetry in Gaussian chains observed by Lapolla and Godec [12], thus obtaining a simplified proof of a physically relevant relaxation asymmetry. Moreover, this example shows that our criterion can be applied beyond the dually flat case.

This work is a continuation of the conversation between geometry and dynamics that Fujiwara and Amari started, where stochastic processes become geodesic motions and where the question of why warming differs from cooling finds a geometric answer. We have organized it as follows. First, we review previous results in Section 2. Then, we present our main results in Section 3 and Section 4. In Section 5, we apply this criterion to Gaussian chains, which are systems of particular physical relevance, and share our conclusions and perspectives in Section 6.

## 2. Gradient Flows on Dually Flat Manifolds

We review here previous results to provide some context for our own. In what follows, $\left(M,g,\nabla ,\nabla^{*}\right)$ denotes an *n*-dimensional dually flat manifold, meaning that

$$X\left[g\left(Y,Z\right)\right]=g\left(\nabla_{X}Y,Z\right)+g\left(Y,\nabla_{X}^{*}Z\right),$$

with both ∇ and $\nabla^{*}$ flat and symmetric.

The metric on a dually flat manifold is special, since it can locally be written as the Hessian of a function ϕ:

$$g_{ij}=\frac{\partial^{2}\phi }{\partial \theta^{i}\partial \theta^{j}}.$$

This is why dually flat manifolds are also known as Hessian manifolds. The θ are affine parameters of ∇, that is, the Christoffel symbols of ∇ in these coordinates are zero.

There is something analogous for the affine parameters η of the dual connection $\nabla^{*}$:

$$g^{ij}=\frac{\partial^{2}\psi }{\partial \eta_{i}\partial \eta_{j}},$$

which are also the components of the inverse matrix of $\left(g_{ij}\right)$. The two metric potentials ϕ(θ) and ψ(η) are related via a Legendre transform:

$$\phi \left(\theta \right)+\psi \left(\eta \right)=\theta^{i}\eta_{i}.$$

Amari and Nagaoka [18] proved that, to any flat connection, we may associate its ∇-divergence a non-negative two-point function on *M* that vanishes only on the diagonal, given by

$$D_{\nabla }\left(p||q\right):=\phi \left(p\right)+\psi \left(q\right)-\theta^{i}\left(p\right)\eta_{i}\left(q\right).$$

When *M* is the parameter space of an exponential family of distributions (which is dually flat with the Fisher metric and the so-called exponential and mixture connections), $D_{\nabla }$ coincides with the Kullback–Leibler divergence between two distributions.

The remarkable property of $D_{\nabla }$ that Fujiwara and Amari [4] proved is that its gradient flows are “straight lines”, as seen by ∇ and $\nabla^{*}$. To be more precise, if we let

$$D_{q}:=D_{\nabla }\left(q||\cdot \right)\;\mathrm{and}\;D_{q}^{*}:=D_{\nabla }\left(\cdot ||q\right)\;,$$

then

$$\nabla_{\mathrm{grad}\;D_{q}}\mathrm{grad}D_{q}=\mathrm{grad}D_{q}\;\mathrm{and}\;\nabla_{\mathrm{grad}\;D_{q}^{*}}^{*}\mathrm{grad}D_{q}^{*}=\mathrm{grad}D_{q}^{*}.$$

That property allowed us to portray Newton’s Law of Cooling as a gradient flow in a previous work [17]. There, we also showed that the non-metricity tensor of the connections, known as the Amari–Chentsov tensor in this context, is instrumental in determining which of two gradient descent curves will reach equilibrium first. Specifically, let $\gamma^{1}$ and $\gamma^{2}$ be two gradient descent curves (that is, two integral curves of $-\mathrm{grad}D_{q}$) starting at the same distance to *q* as measured by $D_{q}$ (namely, such that $D_{q}\left(\gamma^{1}\left(0\right)\right)=D_{q}\left(\gamma^{2}\left(0\right)\right)$). If $\nabla g\left({\dot{\gamma }}_{1},{\dot{\gamma }}_{1},{\dot{\gamma }}_{1}\right)<\nabla g\left({\dot{\gamma }}_{2},{\dot{\gamma }}_{2},{\dot{\gamma }}_{2}\right)$ whenever $\left|\right|{\dot{\gamma }}^{1}{\left|\right|}_{g}=\left|\right|{\dot{\gamma }}^{2}{\left|\right|}_{g}$, then $\gamma_{1}$ will always be closer to *q* than $\gamma_{2}$, i.e., $D_{q}\left(\gamma_{1}\left(t\right)\right)<D_{q}\left(\gamma_{2}\left(t\right)\right)$ for all t>0.

Not much has been done in the case where ∇ (and hence $\nabla^{*}$) is not flat. Matumoto proved [19] that a dual manifold has infinitely many associated divergences, in the sense that the metric and the dual connections can be derived from them (as is the case of the ∇-divergence). Ciaglia et al. proposed a Hamilton-Jacobi approach to define a natural divergence from a dynamical point of view [20]. They also argued that the non-dually flat case is extremely important, since the space of pure states of a finite-level quantum system does not admit a dually flat structure. Following a different path, Amari and Ay found a generalized “canonical divergence” that reduces to $D_{\nabla }$ in the flat case [11], but misses other nice properties like satisfying $D\left(p||q\right)=D^{*}\left(q||p\right)$, or having gradient flows follow pregeodesics.

This coincidence of gradient curves and pregeodesics is precisely what gave the Amari–Chentsov tensor its significance in our asymmetry criterion. It is also sufficient to compare relaxation speeds on any Riemannian manifold: if we can find a connection whose pregeodesics are the gradient curves of a function, then a similar criterion using the non-metricity tensor becomes possible, regardless of flatness. In the next section, we prove that such a connection always exists.

## 3. Gradient Flows Follow Pregeodesics on Riemannian Manifolds

The result of Fujiwara and Amari establishes that, on a dually flat manifold, the gradient flow of a canonical divergence coincides with pregeodesics of its associated flat connection. Can we always find a connection that makes the gradient curves of a given function pregeodesic on an arbitrary Riemannian manifold? We answer by constructing such a connection explicitly.

Let *f* be a smooth function on a Riemannian manifold (M,g). We seek a connection $\nabla^{f}$ for which every gradient descent curve of *f* is a pregeodesic, i.e.,

(1) $$\nabla_{\mathrm{grad}f}^{f}\mathrm{grad}f=\lambda \mathrm{grad}f,$$

for some real function λ.

A good starting point is writing ${\tilde{\nabla }}_{X}^{f}Y=\nabla_{X}^{g}Y-A\left(X,Y\right)Z$, where $\nabla^{g}$ is the metric connection, *A* is a (0,2)-tensor, and *Z* is some vector field to be determined. We can even make it symmetric by choosing *A* symmetric, and a straightforward guess with what we have at hand is A(X,Y)=g(X,Y). Then, the only missing ingredient is *Z*, which we may obtain from Equation (1):

(2) $$\nabla_{\mathrm{gradf}}^{g}\mathrm{grad}f-{\left||\mathrm{grad}f|\right|}^{2}Z=\lambda \mathrm{grad}f.$$

This equation determines *Z* uniquely wherever grad f ≠ 0. Consequently, we obtain the following general result:

**Theorem** **1.** *For any function f, there exists a symmetric connection ${\tilde{\nabla }}^{f}$ defined on M minus the critical points of f by*

(3) $${\tilde{\nabla }}_{X}^{f}Y:=\nabla_{X}^{g}Y-g\left(X,Y\right)\frac{\nabla_{\mathrm{grad}f}^{g}\mathrm{grad}f-\lambda \mathrm{grad}f}{{\left||\mathrm{grad}f|\right|}^{2}},$$

*for some real function λ and satisfying Equation (1). Hence, every gradient curve of f is a pregeodesic of ${\tilde{\nabla }}^{f}$.*

Pripoae and collaborators studied the more general problem of finding a connection for which a given vector field becomes parallel (see [21,22] and the references therein). In contrast, our Theorem 1 provides an explicit construction for the special case of non-singular gradient fields, which does not rely on partitions of unity or the Rectification Lemma. Regardless of how they are obtained, we define them as follows.

**Definition** **1.** *We call any connection $\nabla^{f}$ satisfying Equation (1) a straightening connection of f. Equivalently, we say that $\nabla^{f}$ straightens f.*

The connection constructed in Theorem 1 is not unique. As Pripoae and collaborators showed [22], the set of all connections for which a given non-singular vector field is auto-parallel is an affine space of infinite dimension. Our construction provides only a particular element. Other choices are possible. Importantly, the choice does not affect the asymmetry criterion that we develop in the following sections.

Notice that in the definition of ${\tilde{\nabla }}^{f}$, we may take λ≡0, so that gradf is actually a geodesic of the connection. This will be useful to simplify some calculations later.

It is important to remark that, although every gradient curve of *f* is a pregeodesic of ${\tilde{\nabla }}^{f}$, the converse is not true. Indeed, at any point x∈M, there are infinitely many geodesics (one for each tangent direction), while there is only one gradient curve.

When *f* is the canonical divergence $D_{\nabla }\left(q||\cdot \right)$ of a flat connection (or its dual), the connection ${\tilde{\nabla }}^{f}$ constructed above generally does not coincide with ∇. However, its defining condition given by Equation (1) is the essential property for the asymmetry criterion we will develop later, where the non-metricity tensor is the main character. Actually, the non-metricity tensor

$${\tilde{C}}^{f}\left(W,X,Y\right):={\tilde{\nabla }}_{W}^{f}g\left(X,Y\right)$$

need not be totally symmetric. A direct calculation gives

(4) $${\tilde{C}}^{f}\left(W,X,Y\right)=g\left(W,X\right)g\left(Y,Z\right)+g\left(W,Y\right)g\left(X,Z\right),$$

where *Z* solves Equation (2). This lack of full symmetry contrasts with the Amari–Chentsov tensor, and illustrates how our framework goes beyond the scope of statistical manifolds (including dually flat manifolds).

Theorem 1 provides a recipe to straighten any function *f*. This can be viewed as a partial answer to an inverse problem: while the classical result of Fujiwara and Amari constructs gradient flows that are geodesics given a dually flat structure, our theorem allows us to derive a connection from a function, so that the pregeodesic property of gradf is automatically guaranteed. In the special case where *f* has a unique global minimum *q*, the shifted function $\tilde{f}\left(x\right):=f\left(x\right)-f\left(q\right)$ is always non-negative and vanishes only at *q*. This makes it tempting to consider $\tilde{f}$ as “generating” the dualistic structure $\left(M,g,{\tilde{\nabla }}^{f},{\left({\tilde{\nabla }}^{f}\right)}^{*}\right)$. This perspective is reminiscent of Matumoto’s approach [19], who showed that any statistical manifold can be derived from a divergence. Our work could then extend this idea to a broader setting, where the function *f* is not necessarily a divergence, but induces a connection that straightens it.

In dually flat manifolds, for any submanifold *S*, the ∇-geodesic from a point *q* to a minimizer $\hat{p}\in S$ of the canonical divergence $D_{q}$ meets *S* orthogonally. Thus, minimization of $D_{q}$ over *S* coincides with orthogonal projection along ∇-geodesics. The later extension of canonical divergences by Ay and Amari obeys this so-called geodesic projection property under two assumptions: uniqueness of ∇-geodesics connecting any two points, and the condition that the inverse exponential map at *q* be proportional to $\mathrm{grad}D_{q}$ [11].

**Figure 1:**
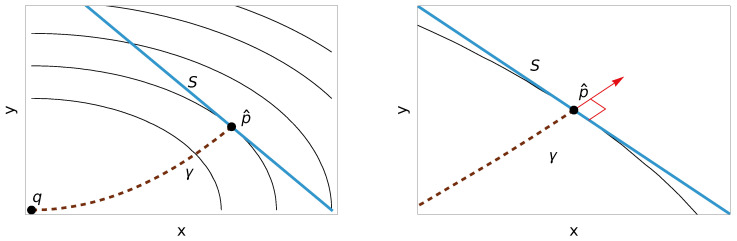
Geodesic projection property. The $\nabla^{f}$-geodesic γ from *q* to $\hat{p}$ (**left**) meets the submanifold *S* orthogonally at the minimizer $\hat{p}$ of *f* on *S* (**right**).

The same holds for every $\nabla^{f}$ whose geodesics connecting any two points are unique, because any connection that straightens *f* has an inverse exponential map proportional to gradf (see Figure 1). We provide an explicit proof below.

**Proposition** **1.** *Let $\nabla^{f}$ be a connection that straightens f, such that the geodesic connecting any two points is unique. Then, $\nabla^{f}$ has the geodesic projection property.*

**Proof.**  If ɩ:S↪M is a submanifold and f∘ɩ attains a minimum at $\hat{p}\in S$, then

$$d{\left(f\circ ɩ\right)}_{\hat{p}}=0.$$

This means that for any vector *v* tangent to *S* at $\hat{p}$,

$$0=d{\left(f\circ ɩ\right)}_{\hat{p}}\left(v\right)=df_{\hat{p}}\left(v\right)=g_{\hat{p}}\left(\mathrm{grad}f_{\hat{p}},v\right),$$

where we are denoting by the same $\hat{p}$ and *v* their images under ı and $d{ɩ}_{\hat{p}}$, respectively.

Finally, since the $\nabla^{f}$-geodesic connecting *q* and $\hat{p}$ is unique, its velocity must be proportional to grad f because the connection straightens *f*, and so the geodesic meets *S* orthogonally at $\hat{p}$.      □

In what follows, we will use Theorem 1 to extend our previous characterization of asymmetric relaxations [17] to general Riemannian manifolds.

## 4. Asymmetric Relaxations on Riemannian Manifolds

In previous work, we showed that on dually flat manifolds, the component of ∇g along gradient descent curves of the canonical divergence determines which of two such curves relaxes first, provided that both start equally “far” from *q* [17]. There, we measured distance with the canonical divergence itself, and we used the fact that its gradient descent curves are ∇-pregeodesics. This fact alone suffices to obtain an analogous asymmetry criterion in a much broader setting.

Before stating our result, notice that considering gradient descent curves γ relaxing to the unique minimum *q* of a function *f* provides a natural notion of “distance” to *q*, given by f(γ(t)), assuming, without loss of generality, that f(q)=0. This motivates the following definition.

**Definition** **2.** *Let $\gamma_{1}$ and $\gamma_{2}$ be two integral curves of the gradient descent of f. We say that their initial conditions are f-equidistant from q, or simply f-equidistant, if $f\left(\gamma_{1}\left(0\right)\right)=f\left(\gamma_{2}\left(0\right)\right)$. Moreover, if $f\left(\gamma_{1}\left(t\right)\right)\leq f\left(\gamma_{2}\left(t\right)\right)$ for all t≥0, then we say that $\gamma_{1}$ is faster than $\gamma_{2}$.*

Now we can state our second main result.

**Theorem** **2.** *Let f be a real function on M attaining its unique minimum at q. Let $\nabla^{f}$ straighten f with λ≡const. Let $\gamma_{1}$ and $\gamma_{2}$ be two integral curves of the gradient descent of f with initial conditions f-equidistant from q. If*

(5) $$C^{f}\left({\dot{\gamma }}_{1},{\dot{\gamma }}_{1},{\dot{\gamma }}_{1}\right)<C^{f}\left({\dot{\gamma }}_{2},{\dot{\gamma }}_{2},{\dot{\gamma }}_{2}\right),$$

*whenever $\left|\right|{\dot{\gamma }}_{1}\left|\right|=\left|\right|{\dot{\gamma }}_{2}\left|\right|$; then, $\gamma_{1}$ is faster than $\gamma_{2}$.*

**Proof.**  We want to see that, with the above hypotheses, $\Delta f:=f\left(\gamma_{2}\left(t\right)\right)-f\left(\gamma_{1}\left(t\right)\right)\geq 0$, for all t≥0. Given that *f* is sufficiently well behaved, this would follow if all critical values of Δf are maxima, since $\Delta f\left(0\right)=\lim_{t\to \infty }\Delta f=0$. Denote by γ an integral curve of the gradient descent of *f*. Then, $\dot{\gamma }=-\mathrm{grad}\;f$ and

(6) $$\dot{f}=df\left(\dot{\gamma }\right)=g\left(\mathrm{grad}f,\dot{\gamma }\right)=-\left|\right|\dot{\gamma }{\left|\right|}^{2}.$$

So, when $\left|\right|{\dot{\gamma }}_{1}\left|\right|=\left|\right|{\dot{\gamma }}_{2}\left|\right|$, Δf attains a critical value, whose nature is determined by the sign of $\Delta \ddot{f}.$ From the last equation, we have that $\lambda \Delta \dot{f}\left(t_{*}\right)=0$, and thus

(7) $$\begin{array}{cc} \ddot{f}=-\dot{\gamma }\left[g\left(\dot{\gamma },\dot{\gamma }\right)\right] & =-\nabla_{\dot{\gamma }}^{f}g\left(\dot{\gamma },\dot{\gamma }\right)-2g\left(\nabla_{\dot{\gamma }}^{f}\dot{\gamma },\dot{\gamma }\right)\\ \; & =-C^{f}\left(\dot{\gamma },\dot{\gamma },\dot{\gamma }\right)+2\lambda g\left(\dot{\gamma },\dot{\gamma }\right)=-C^{f}\left(\dot{\gamma },\dot{\gamma },\dot{\gamma }\right)-2\lambda \dot{f}, \end{array}$$

using Equation (1) in the third equality. Then,

(8) $$\Delta \ddot{f}\left(t_{*}\right)=-C^{f}\left({\dot{\gamma }}_{2},{\dot{\gamma }}_{2},{\dot{\gamma }}_{2}\right){|}_{t_{*}}+C^{f}\left({\dot{\gamma }}_{1},{\dot{\gamma }}_{1},{\dot{\gamma }}_{1}\right){|}_{t_{*}}-2\lambda \left(\gamma_{2}\left(t_{*}\right)\right)df\left({\dot{\gamma }}_{2}\right){{|}_{t_{*}}+2\lambda \left(\gamma_{1}\left(t_{*}\right)\right)df\left({\dot{\gamma }}_{1}\right)|}_{t_{*}}$$

Using Theorem 1, we can choose a connection $\nabla^{f}$ that straightens *f* with λ≡const., so that the last two terms are proportional to $\Delta \dot{f}\left(t_{*}\right)=0$. This way, the last expression is negative if and only if

(9) $$C^{f}\left({\dot{\gamma }}_{1},{\dot{\gamma }}_{1},{\dot{\gamma }}_{1}\right){{|}_{t_{*}}<C^{f}\left({\dot{\gamma }}_{2},{\dot{\gamma }}_{2},{\dot{\gamma }}_{2}\right)|}_{t_{*}},$$

and $\Delta f(t_{*})$ is a maximum.

Thus, if (9) holds every time the descent speeds are equal, the relaxation along $\gamma_{1}$ will be faster than that along $\gamma_{2}$.     □

Equation (7) expresses $C^{f}\left(\dot{\gamma },\dot{\gamma },\dot{\gamma }\right)$ in terms of *f* and λ only. Consequently, any straightening connection of *f* shares this component of its non-metricity along the gradient flow. In particular, a connection $\nabla^{f}$ of which the gradient curves are geodesics (λ≡0) simplifies Equation (7) to

(10) $$\ddot{f}=-C^{f}\left(\dot{\gamma },\dot{\gamma },\dot{\gamma }\right)\;.$$

If we regard geodesics as motions of “free particles” in analogy with classical mechanics, then $-\ddot{f}$ plays the role of an effective force that drives the system toward the minimum of *f*. For a metric connection ($C^{f}\equiv 0$), this force vanishes and the descent is uniform. Non-metricity thus measures how the geometry encoded in the connection accelerates the particle along its path. When two gradient curves have the same speed at some instant, the one with the more negative $C^{f}$ experiences a greater driving force, making its speed increase at a higher rate and consequently reach the minimum faster.

We may also express the non-metricity tensor along gradient curves in terms of the curves. Using the Levi-Civita connection,

$$\ddot{f}=-2g\left(\nabla_{\dot{\gamma }}^{g}\dot{\gamma },\dot{\gamma }\right).$$

Substituting into (7) yields an equivalent formula that involves only γ:

(11) $$C^{f}\left(\dot{\gamma },\dot{\gamma },\dot{\gamma }\right)=2\left[\lambda ∥\dot{\gamma }{∥}^{2}+g\left(\dot{\gamma },\nabla_{\dot{\gamma }}^{g}\dot{\gamma }\right)\right]$$

**Figure 2:**
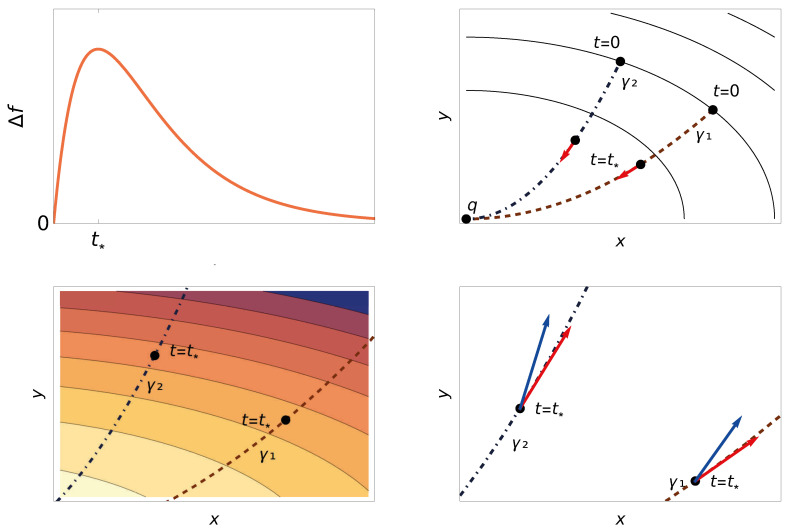
An illustration of our asymmetry criterion. (**Upper left**): $\Delta f\left(t\right)=f\left(\gamma_{1}\left(t\right)\right)-f\left(\gamma_{2}\left(t\right)\right)$ attains its maximum at $t=t_{*}$. (**Upper right**): Two gradient descent curves $\gamma_{1}$ and $\gamma_{2}$ with *f*-equidistant initial conditions. At $t_{*}$, their velocities (red) have equal length. (**Lower left**): Heat map of $-C^{f}\left(\mathrm{grad}f,\mathrm{grad}f,\mathrm{grad}f\right)$ (lighter = larger). At $t_{*}$, $C^{f}\left(\gamma_{1},\gamma_{1},\gamma_{1}\right)>C^{f}\left(\gamma_{2},\gamma_{2},\gamma_{2}\right)$, so $\gamma_{2}$ relaxes faster. (**Lower right**): Covariant acceleration (blue) at $t_{*}$. Its tangential component (red) is negative for both curves, larger in magnitude for $\gamma_{2}$, explaining its faster relaxation.

Thus, Theorem 2 states that the curve having the smallest tangential metric acceleration when the relaxation speeds coincide will always be faster (see Figure 2).

Our asymmetry criterion also tells us when asymmetry is forbidden.

**Corollary** **1.** *If f is such that one of its straightening connections is a metric connection with λ≡const., then the gradient curves of f cannot show any asymmetry.*

**Proof.**  If $\nabla^{f}$ is metric, Equation (7) reduces to $\ddot{f}=-2\lambda \dot{f}$, and therefore we have, for all *t*,

$$\Delta \ddot{f}=-2\lambda \Delta \dot{f}\;.$$

If λ≡const., the conditions $\Delta f\left(0\right)=\lim_{t\to \infty }\Delta f=0$ force Δf≡0. □

A straightforward consequence of this is that the Riemannian distance function does not have asymmetric relaxations.

However, this result is more interesting when read in the opposite direction: functions for which a relaxation asymmetry exists cannot be straightened by a metric connection (with λ≡const.).

Symmetry, however, does not necessarily imply metricity, because asymmetric relaxations are determined by the function *f* only, not by the connection used to determine them. Indeed, we can use Theorem 1 to find non-metric connections that straighten a function whose relaxation is symmetric.

With Equation (3), we can illustrate how Theorem 2 works explicitly on a manifold that is not dually flat, which is also of physical relevance.

## 5. Universal Asymmetry in Relaxations of Gaussian Chains

Using our criterion, we can now prove the asymmetry in the relaxation of Gaussian chains reported by Lapolla and Godec [12]. In this context, the distance to equilibrium will be measured by the gradient potential of the dynamics, which is related to the dual Kullback–Leibler divergence.

Gaussian chains are N+1 beads connected by ideal springs with zero rest length. The dynamics of their normal modes are described by multivariate Gaussian distributions with zero means and variances $a_{k}$, which evolve according to

(12) $$a_{k}=2\frac{1+\left(\tilde{T}-1\right)e^{-2\lambda_{k}t}}{\lambda_{k}}.$$

The $\lambda_{k}$ are the eigenvalues of the matrix that allows us to work with independent modes. The parameter $\tilde{T}$ denotes the ratio between the initial temperature of the system and the equilibrium temperature (see Supplementary Material of Ref. [12]).

An interesting feature of Gaussian chains is that they display a universal asymmetry: a system warming up ($\tilde{T}<1$) reaches equilibrium faster than one cooling down ($\tilde{T}>1$) when both start equally far from equilibrium [12], with the distance measured by the KL divergence. In what follows, we reproduce a similar result based on Theorem 2.

Equation (12) is the solution of

(13) $${\dot{a}}_{k}=-2\lambda_{k}\left(a_{k}-a_{k}^{*}\right),$$

with initial condition $a_{k}\left(0\right)=2\tilde{T}/\lambda_{k}$ and $a_{k}^{*}=2/\lambda_{k}$.

This is the gradient flow of

$$F:=\sum_{k}^{N}\lambda_{k}\left(\frac{a_{k}^{*}}{a_{k}}-\ln\left(\frac{a_{k}^{*}}{a_{k}}\right)-1\right).$$

The metric here is the Fisher metric for multivariate normal distributions, which is block-diagonal, with each block corresponding to the Fisher metric of a single mode:

$$g_{k}=\frac{2}{a_{k}}d\mu_{k}^{2}+\frac{1}{2a_{k}^{2}}da_{k}^{2}\;;$$

the gradient potential *F* is proportional to the weighted sum of the (mixture) KL divergence of each mode,

$$D_{\mathrm{KL}}(a_{k}^{*}\left|\right|a_{k})=\frac{1}{2}\left[\frac{a_{k}^{*}}{a_{k}}-\ln\left(\frac{a_{k}^{*}}{a_{k}}\right)-1\right].$$

Since the gradient potential has a global minimum at $\left(a_{1}^{*},\ldots ,a_{N}^{*}\right)$, we can use it as a measure of distance to equilibrium.

To apply our criterion in Theorem 2, we will first consider one mode of the chain. All the modes are independent, so the dynamics given by Equation (13) is still a gradient flow, with gradient potential $F_{k}:=2\lambda_{k}D_{\mathrm{KL}}(a_{k}^{*}\left|\right|a_{k})$.

Using a connection that straightens $F_{k}$ with λ≡0 (see Equation (10)) we obtain

$$-C^{F_{k}}\left(\dot{\gamma },\dot{\gamma },\dot{\gamma }\right)={\ddot{F}}_{k}=2\lambda_{k}\frac{a_{k}^{*}}{a_{k}}{\left(\frac{{\dot{a}}_{k}}{a_{k}}\right)}^{2},$$

for any gradient descent curve of $F_{k}$.

Now, let $\gamma^{\pm }=\left(0,a_{k}^{\pm }\right)$ be two such curves, which are also solutions of Equation (13) given by Equation (12), having initial temperatures ${\tilde{T}}^{+}>1$ and ${\tilde{T}}^{-}<1$, respectively. At the critical point $t_{*}$ of $\Delta F_{k}$, the speeds of $a_{k}^{+}$ and $a_{k}^{-}$ must be the same, meaning that

$${\left(\frac{{\dot{a}}_{k}^{+}}{a_{k}^{+}}\right)}_{t_{*}}^{2}={\left(\frac{{\dot{a}}_{k}^{-}}{a_{k}^{-}}\right)}_{t_{*}}^{2}=:S\;.$$

Plugging this into $\Delta {\ddot{F}}_{k}{|}_{t_{*}}$ we obtain

$$\Delta {\ddot{F}}_{k}{|}_{t_{*}}=-C^{F_{k}}{\left({\dot{\gamma }}^{+},{\dot{\gamma }}^{+},{\dot{\gamma }}^{+}\right)}_{t_{*}}+C^{F_{k}}{\left({\dot{\gamma }}^{-},{\dot{\gamma }}^{-},{\dot{\gamma }}^{-}\right)}_{t_{*}}=2\lambda_{k}S{\left(\frac{a_{k}^{*}}{a_{k}^{+}}-\frac{a_{k}^{*}}{a_{k}^{-}}\right)}_{t_{*}}<0\;,$$

because $\lambda_{k}>0$ and $a_{k}^{-}<a_{k}^{*}<a_{k}^{+}$ always. So, $\gamma^{-}$ is faster than $\gamma^{+}$, according to Theorem 2. Since this is true for every mode, $\sum \Delta F_{k}=\Delta F\geq 0$, and therefore the chain warms up faster than it cools down.

To make sure that we are truly beyond the dually flat case, we may compute the curvature tensor of ${\tilde{\nabla }}^{F_{k}}$ with λ≡0. One of its components is

$$R_{122}^{1}=-\frac{a_{k}-3a_{k}^{*}}{4a_{k}^{2}\left(a_{k}-a_{k}^{*}\right)}$$

This example works as a consistency check for our criterion in Theorem 2. It correctly reproduces a well-known physical asymmetry, and also reveals that this effect holds for a different choice of distance measure (the dual KL divergence). Any curve starting below the equilibrium temperature will relax faster than any other curve starting above it, provided that they start from *F*-equidistant initial conditions. This is parameterized by $\tilde{T}$ and, in this sense, the asymmetry is universal, and is simply stated as warming up is faster than cooling down.

Similar asymmetries take place in driven classical systems [23] and in open quantum systems [15], where heating is also faster than cooling. Those studies rely on matrix analysis or quantum information measures, while our geometric criterion identifies the non-metricity tensor as the key quantity determining the asymmetry. The occurrence of this physical effect across driven and quantum regimes suggests a universal feature of relaxation, possibly rooted in the geometric structure of the space of states.

## 6. Concluding Remarks

Showing the deep connection between gradient flows and pregeodesics in Hessian manifolds was part of Amari’s valuable contribution to information geometry. Besides the many implications and applications of this insight, it was a fundamental component of the geometric criterion for asymmetric relaxations that we developed previously [17]. In that context, the distance to equilibrium is measured by the KL divergence, which makes sense in information geometry and thermodynamics. We extended this result to Riemannian geometry, considering that any function with a unique minimum works as well.

In dually flat manifolds, it is fundamental that the connection we use to define geodesics is flat, because pregeodesics and gradient descents of the canonical divergence coincide in that case [4]. However, this is no longer true for any divergence function, nor for the “canonical” divergence of a non-flat connection [11]. We translate this obstacle to finding a connection whose pregeodesics describe a given gradient, and we provide an explicit recipe for this in Theorem 1. Of course, many more connections that straighten a given *f* with other desirable properties may exist. Their characterization is an interesting open problem that might provide new insights to generalize canonical connections in information geometry.

There are many directions in which our result may be extended. One is to consider dynamics that are not necessarily gradient flows, for which the function *f* in Theorem 1 is a Lyapunov function. Another is to understand if symmetry in relaxation always implies the existence of a metric connection that straightens *f*.

Furthermore, our geometric characterization of asymmetric relaxations on Riemannian manifolds can be useful in optimization problems where only the initial *f*-distance to a minimum is known. Theorem 2 may help to choose an initial condition that will make the gradient descent faster.

Finally, the geometric framework developed here provides a natural setting to investigate further instances of asymmetric relaxations in thermodynamics. In particular, the Mpemba effect, both classical and quantum, offers a fertile ground for the application of these ideas [24,25,26,27,28]. Our reproduction of the asymmetric relaxation in Gaussian chains using a distance measure that is different from the Kullback–Leibler divergence also aligns with recent work that points to a divergence-independent characterization of relaxation asymmetries [29].

These perspectives speak to the potential of the nice interplay between geometry and dynamics first illuminated by Amari and his collaborators.

## Data Availability

No new data was created.
